# Perceptions and Patterns of Dietary Supplements’ Use during COVID-19 among Undergraduate Female Students in Saudi Arabia

**DOI:** 10.3390/nu14183728

**Published:** 2022-09-09

**Authors:** Wafa Hamad Almegewly, Rimah Bader Alenazi, Fayhaa Mohammed Albaqami, Raghad Abdulkarim Alkharashi, Fatimah Abdulrhman Alsaedi, Reem Khalaf Almutairi, Alhanouf Abdullah Alkharji, Ghadeer Mohammed Althani, Wafa Abdullah Aljuwayd

**Affiliations:** 1 Department of Community Health Nursing, College of Nursing, Princess Nourah Bint Abdulrahman University, P.O. Box 84428, Riyadh 11671, Saudi Arabia; 2 College of Nursing, Princess Nourah Bint Abdulrahman University, P.O. Box 84428, Riyadh 11671, Saudi Arabia

**Keywords:** COVID-19, dietary supplements, multivitamin, university, students, knowledge, pattern

## Abstract

(1) Background: During the COVID-19 pandemic, the use of Dietary Supplements (DSs) has increased for health promotion purposes. Few data records were found on the safe use of DSs among university students in Saudi Arabia, during COVID-19. This study aimed to assess the perceptions and patterns of DSs during COVID-19 among students at a selected female university. (2) Methods: A descriptive cross-section design was used. A convenient sample of undergraduate students (*n* = 651) were recruited via email, to fill in an online validated questionnaire: The nutrition and intake of DSs during COVID-19. Data were analyzed using descriptive and inferential statistics. (3) Results: Among the 509 students who did not have chronic diseases, 85% of them had taken DSs. About 35.5% of the students had not changed their dietary habits since the beginning of COVID-19, but 78.6% thought that they needed to improve their immunity by taking DSs. Half of the students 51.2% believed that healthy habits may reduce the chance of being infected with COVID-19. The most used DSs were vitamin C (84.3%), followed by honey (65.3%), and vitamin D (47.7%). At the top of students’ references for DSs was personal judgment or previous knowledge of the benefits (27.3%). (4) Conclusion: The usage and patterns of DSs were impacted by the COVID-19 pandemic. Taking DSs without a doctor’s prescription may lead to several complications. DSs users should be educated effectively about the proper use of DSs as an external supplementation.

## 1. Introduction

The global outbreak of the COVID-19 pandemic has spread around the world, affecting almost every country and region. The outbreak was first confirmed in Wuhan, China in December 2019. According to the World Health Organization (WHO), COVID-19 is an infectious disease caused by the SARS-CoV-2 virus. On 31 December 2019, the coronavirus was eventually identified. Globally, by 15 June 2022, there were 534,495,291 confirmed cases of COVID-19, including 6,311,088 deaths [1]. As of 6 June 2022, a total of 11,864,214,773 vaccine doses have been administered [1]. Meanwhile, in Saudi Arabia the first case of coronavirus infection was announced on 2 March 2020 according to the Ministry of Health (MOH). From 3 January 2020 to 15 June 2022, there were 780,135 confirmed cases of COVID-19 with 9176 deaths, and by 31 May 2022, a total of 65,837,671 vaccine doses had been administered [2]. Since the COVID-19 pandemic started, the sales of dietary supplements increased dynamically across the world. The market for supplements in the United Kingdom increased by 19.5%, especially during the period of the “lockdown” in early March 2020 [3]. Some types of DSs even recorded triple-digit growth rates (Euromonitor International, 2020), such as vitamins C and D, and zinc [4]. Worldwide and particularly in Saudi Arabia, many people went to protect themselves by taking DSs, specifically vitamin C [5]. The consumption of DSs and herbal products increased by 22.1% among the Saudi population; additionally, 29.3% of participants think that the consumption of vitamin C has a role in treating or reducing the chances of developing COVID-19 infection [6]. In the Middle East, there was a noticed change in DSs intake patterns during the pandemic, as 70% of the participants believed healthy habits may help in preventing infections [7]. In Lebanon, the consumption of DSs containing vitamin C during COVID-19 increased from 35.3% to 42.1%, and vitamin D from 35.5% to 41%, while other supplements, such as vitamin E, zinc, omega-3, garlic, ginger, turmeric, and other vitamins, increased from 9% to 10.9% [8]. In Poland, the usage of DSs during the first wave of COVID-19 was 79% and decreased in the second wave to be 60%. They added that during the early stages of the pandemic, several types of DSs even had triple-digit growth rates because of the high demands to improve immunity and the limited broad availability of COVID-19 vaccines [4]. In addition, in the US, the usage of DSs before the pandemic was 40.6%, and during the pandemic, it became 75.7% [9].

Participants’ choice to consume DSs depends on several factors such as gender, lifestyle, age, family income, presence of pregnancy, and health status. It was confirmed that university students, young women aged 18–24 years, consumed supplements at a higher rate than other age groups [10]. It was found that the use of DSs and herbal products, such as garlic and cinnamon, increased more among women than men before the COVID-19 pandemic period [11]. In addition, in Italy over-the-counter supplements were commonly used by young women to protect their health [12]. Concerning the lifestyle aspect, the undergraduate student belief that most influences their use is disease prevention (70.9%), followed by disease control and treatment (68.5%), then fat loss (62.1%), and student athletes reported using DSs for disease treatment (82.6%), for disease prevention (80.8%), for physical performance (71.9%), and both health and wellness and muscle building were equal (66.7%) [13]. In addition, another study found that family income has no effect on the usage of DSs [14].

The studies showed that the level of knowledge about DSs is quite low, especially among university students. Consulting a physician before taking supplements is essential, as unnecessary supplement use can be harmful to health [15]. On the other hand, social media and the Internet (29.7%), as well as relatives or friends (14.7%), were the top motivators for people to try herbal remedies [11]. These findings are comparable to those of a recent cross-sectional study, which indicated that social media and the Internet were the leading sources of recommendations for participants to consume DSs and herbal products (39.4%) [6]. It was evident that 38.5% of the Saudi participants considered that DSs may cause harm, while others, by 57.8%, believed that they should be routinely included in their diet [16]. Another study found that 89.9% of the participants defined supplements correctly, and more than half of the sample believed that supplements are safe [17]. On the other hand, the excessive use of DSs without medical supervision could lead to nausea, diarrhea, stomach cramps, hair loss, and fatigue [16].

There are several studies completed in Saudi Arabia, assessing the knowledge and patterns of DSs among the normal population (16–19). However, few studies were conducted measuring the association between COVID-19 and the usage of DSs [5,6,18]. The sample used for the latter studies was the normal population and patients, but none of them were female university students since they are the most common consumers of DSs. This study aims to assess the perceptions and patterns of DSs during COVID-19 among undergraduate students.

## 2. Materials and Methods

### 2.1. Research Designs

This is a descriptive cross-sectional design.

### 2.2. Setting and Sampling

The data were collected from 16 colleges at the selected female university in Riyadh city, Saudi Arabia. The study population is undergraduate students who attend the selected female university (*n* = 98301). The sample size was calculated according to Epi Info calculation, with a margin of error of 5 and a confidence interval of 95%. The estimated calculated sample size is *n* = 383, but in this study, the authors were able to collect data from a convenient sample of *n* = 651.

### 2.3. Data Collection Measurement and Process

The used tool is the “Nutrition & Intake of DSs during the COVID-19” questionnaire [7]. The survey is in English and consists of 26 questions divided into five sections; the first section was demographic information such as age, college, academic year, the student’s GPA, smoking, and exercise status. The second section consisted of the health status. The third section contained chronic diseases, their current medications, and if the participants have been infected with COVID-19 or not. The fourth section asked about the participant’s knowledge about DSs intake in the treatment of COVID-19. The last section included questions about the participant’s usage and intake of dietary supplements during the COVID-19 pandemic to improve their immunity, the main reason to note taking the dietary supplements, the most supplements used during the COVID-19 pandemic, and who suggested that you take dietary supplements.

The data were collected from February to March 2022. The questionnaire was uploaded on Google Forms and distributed via email by the students’ affairs unit of each college to all of the students’ emails. The students were able to complete the questionnaire within 5 min.

### 2.4. Ethical Consideration

This study was ethically approved by the Institutional Review Board (IRB) of the selected university (ref no 22-0088). The participants’ consent to take part in the research was safeguarded online on the first page of the online questionnaire prior to filling it. The study subjects have been advised of the right to withdraw from the study at any time.

### 2.5. Data Analysis

Descriptive and inferential statistics were applied to analyze the data, using the Statistical Package for Social Studies Software (SPSS) version 23. The descriptive statistics, including frequency and percentage, were used to describe and organize the demographic profiles of the participants. Inferential statistics were used to draw inferences about the population including using Pearson Correlation to determine the correlation among the variables and the Chi-square test to check the data for normality, ratio scales to test hypotheses, and examining the relationship between the variables. In all of the tests, a *p*-value < 0.05 was considered statistically significant. The independent variables were selected after their independence was verified. Pearson’s correlation coefficient (*r*) values less than 0.9 indicated that multicollinearity was not present in regression analysis.

## 3. Results

The largest number of students who participated in the study were from the College of Computer and Information Science (15.2%), followed by the College of Science (14%), College of Art (12.7%), College of Business Administration (12.1%), College of Education (11.1%), College of Social Work (6.3%), College of Nursing (5.8%), College of Languages (5.2%), College of Health and Rehabilitation Science (5.1%), College of Law (3.1%), College of Medicine (2.2%), College of Foundation year (1.7%), and the remainder from the Colleges of Dentistry, Pharmacy, and Engineering (Table 1).

In terms of frequency and percentage distribution of health indicators among the respondents, the health indicator practices/presence among the students, almost all (95.5%) of the students do not smoke, 2.6% smoke, and 1.8% are ex-smokers. The majority of the students (56.7%) do not exercise, while 22.9% do so only once a week, 14% do 2–3 times a week, 4.8% 4–5 times a week, and only 1.7% exercise daily. Almost half of them (41.5%) had had the COVID-19 infection. There are 9.4% who have chronic diseases while the majority (90.6%) have none.

Among those with chronic diseases, the majority have bronchial asthma (54.1%), followed by diabetes mellitus (16.4%), hypothyroidism (11.5%), hypertension (6.6%), rheumatism and ulcerative colitis each (3.3%), respectively, and several others. There are 59% of them taking medications while 41% are not taking any medication for chronic diseases. The majority (43.2%) of them self-reported their general health to be very good, 25.7% self-reported as good, 23.7% as excellent, 6% as fair, and 1.4% as a weak general health status (Table 2).

Among the 61 students with chronic diseases, only 18% used DSs during the COVID-19 outbreak, while 82% did not take DSs. Among the 509 students who do not have chronic diseases, 85% of them have taken DSs, and 15% have not (Table 3).

The results show that the students who belong to the health sciences programs are more likely to have better knowledge about DSs than the students who are from the non- health sciences, as shown by the chi-square score of 119.45 with *p*-value < 0.00001which is less than the alpha of 0.05 (Table 4).

Regarding the frequency and percentage distribution of behavior during the pandemic, there were 22.4% of the students who changed their dietary habits for the better during the COVID-19 pandemic, while 18% changed for the worse, 35.5% did not change, and 24.1% cannot specify. The majority (81%) think that they need to improve their immunity by changing some unhealthy dietary habits, 10% think otherwise, while 9.1% do not know. More than half of them (51.2%) think that following healthy habits may reduce their chance of getting Coronavirus, 28.3% think not, and 20.6% do not know (Table 5).

The reasons identified by the respondents for not taking DSs include the highest which is no specific reason (36.9%), followed by cannot take the supplements regularly (29.2%), do not know which are available in the market (22.9%), they are a high price (21.7%), I do not need them/do not have a vitamin deficiency (17.4%), they have some side effects (15.4%), I am committed to a healthy and balanced diet (5.4%), they are unsafe (3.2%), they are ineffective (2.3%), and they interact with home medications (1.6%) (Table 6).

There are 79.9% of students who took vitamin C supplements during the COVID-19 outbreak, 47.4% took honey, 35.9% took vitamin D, 30.1% took iron, and 22.5% took garlic. Other supplements included multivitamins, vitamin B12 complex, Folic acid, Fish oil, and others (Table 7).

There are 19.1% who took suggestions to take the DSs from a physician, 21.9% from a friend/relative, 16.7% from social media/Internet, 5.5% from a pharmacist, and the majority (27.3%) took supplements from their personal judgment or previous experience of the benefits (Table 8).

## 4. Discussion

This study assessed the perceptions and patterns of DSs’ usage during COVID-19 among undergraduate students. It was found that 85% of students with no chronic disease were taking DSs during COVID-19. This is congruent with other studies that revealed that students between the ages of 17 and 30 had the highest prevalence of DSs’ usage [18,19]. There are many possible reasons for this, such as addressing health concerns during exams; combating stress; accomplishing an aesthetic goal or a desire to enhance muscle mass [19]. This study also showed that the students with chronic diseases were only using DSs that included a limited amount of herbal substances. This was in contrast to a previous study by [20], which found that patients with type 2 diabetes utilize herbal supplements and a combination of herbal supplements and DSs. People with chronic diseases may consume fewer DSs because they can either enhance or counteract the effects of prescription medications [21]. In particular, several DSs may interact with over-the-counter drugs [22]. In addition, this study found that the health sciences students were more likely to have better knowledge about DSs than those who were from the non-health sciences colleges. This might be explained by their background in nutrition. The findings of the current study are in contrast with another study that found that 43% of health sciences students were not highly knowledgeable about DSs, as many of them do not have accurate information about the health benefits of supplements [10].

The current study reports that the students who are following healthy habits may reduce the risk factors of becoming infected with COVID-19. These findings are further supported by another study that found that effective habits could aid in preventing infection [7]. The Saudi Ministry of Health’s efforts to broaden the continuing immunization campaign and make vaccine facilities more accessible were reflected in these results. Coronavirus vaccinations were provided as a service for free to all of the Saudi citizens and residents in pharmacies across the country [23]. The study showed that some of the students reported that the high price of DSs was the reason for not taking them which is in contrast with another study that reported that the cost has no significant effect on usage [14]. Others reported there was no specific reason for not taking the DSs, at the same time the majority of students confirmed that they are taking DSs. From participants’ perspectives of the importance of DSs, they believe that vitamin C is the most significant vitamin followed by vitamin D, and honey which may help their immunity against COVID-19. This was reflected in the rate of usage of later supplements during the pandemic as well as in the market growth rate. These findings match with previous studies [23]. The possible explanation for the high intake of DSs during the COVID-19 pandemic is the fear that inhabited the souls of people about the risk of infection with COVID-19 and the lack of treatment, which led to the necessity of protecting oneself using supplements necessary to raise immunity [6]. The six vitamins D, A, C, folate, B6, B12 and the four minerals zinc, iron, copper, and selenium are the most promising for managing COVID-19 symptoms, according to the European Food Safety Authority (EFSA). These have been shown to have a stronger effect on COVID-19 symptoms, prevention, treatment, and management [8].

In terms of reference for DSs, this study found that more than half of the participants reported that they were using DSs without doctor’s prescription. Similar results were reported by [22]. The majority of the participants reported that they were sure that using DSs has side effects, in contrast with another study which reported that 62% of the participants believed that DSs are necessary for their health and could be used without a doctor’s prescription and they are safe [16]. This study also showed that 42% of the students were not sure if taking DSs may help in treating a person infected with COVID-19. This was in contrast to another study that found that 45.4% of the participants reported that using DSs can treat patients with COVID-19 [7].

### 4.1. Strength and Limitations

The key strength of this study is using a large sample size of young participants, which enhanced the sense of generalizability. The fact that COVID-19 is a trending topic until now as the world is still trying to deal with COVID-19 has added to the importance of this study. The current findings add to a growing body of literature on COVID-19 and the usage of DSs.

This study has a few limitations, despite the moderate sample size, only young female perspectives were represented rather than male. This is because the selected setting is a university for women only and that may limit generalizability to the Saudi male community. It is regrettable that diverse age groups, such as elderly adults, were not included in the study. It is recommended that further research be large randomized controlled trials that could provide more definitive evidence. More research is required to determine the DSs’ consumption and the association with people who have chronic diseases.

### 4.2. Research Implications

The results of this study open up new lines of similar research. It is hoped that other researchers will consider examining the impact of COVID-19 on the consumption of DSs. The results of this study can further be validated by considering a wider study that would collect both quantitative and qualitative data to give a deeper understanding of the effects of this epidemic and the usage of DSs. This study declared the importance of raising community awareness by using advertising materials such as TV shows and performing awareness campaigns in the mall and schools.

## 5. Conclusions

This study set out to assess the perceptions and patterns of DSs during the COVID-19 pandemic among undergraduate students. The results of this study indicate that the use of DSs has increased during the pandemic of COVID-19, especially the consumption of vitamin C, as the participants reflect that may improve their immunity and reduce their risks of being infected with COVID-19. The health sciences students have more knowledge about the DSs compared to the others. Moreover, the DSs users are more likely to take supplements on their own instead of consulting with healthcare providers.

## Figures and Tables

**Table 1:** Demographic profile.

Profile	Frequency	Percentage
**Age**		
16–20	322	49.5
21–25	321	49.3
26–30	5	0.8
31–35	3	0.5
**College**		
College of Education	72	11.1
College of Art	83	12.7
College of Languages	34	5.2
College of Science	91	14
College of Computer and Information Science	99	15.2
College of Nursing	38	5.8
College of Social work	41	6.3
College of Dentistry	7	1.1
College of Pharmacy	8	1.2
College of Medicine	14	2.2
College of Business Administration	79	12.1
College of Art and Design	9	1.4
College of Engineering	3	0.5
College of Law	23	3.5
College of Health and Rehabilitation Science	33	5.1
College of Foundation Year	11	1.7
**Academic Year**		
Preparatory	143	22
2nd year	158	24.3
3rd year	120	18.4
4th year	146	22.4
5th year	20	3.1
6th year	36	5.5
Intern	28	4.3
**GPA**		
5.0–4.50	260	39.9
4.49–3.75	321	49.3
3.74–2.75	60	9.2
2.74–2	10	1.5

**Table 2:** Frequency and percentage distribution of health indicators among the respondents.

Health Status Scales	Frequency	Percentage
Smoking		
Yes	17	2.6
No	622	95.5
Ex-smoker	12	1.8
Exercise		
Do not exercise	369	56.7
Exercise once a week	149	22.9
Exercise 2–3 times a week	91	14
Exercise 4–6 times a week	31	4.8
Exercise daily	11	1.7
COVID-19 infection		
Yes	270	41.5
No	381	58.5
Self-report general health status		
Excellent	154	23.7
Very good	282	43.3
Good	167	25.7
Fair	39	6
Weak	9	1.4
Presence of Chronic Disease		
yes	61	9.4
no	590	90.6
Types of Chronic Diseases (those diagnosed)		
Bronchial asthma	33	54.1
Ulcerative colitis	2	3.3
Diabetes mellitus	10	16.4
Hypertension (high blood pressure)	4	6.6
Rheumatism	2	3.3
Hypothyroidism	7	11.5
Hyperthyroidism	1	1.6
Liver failure	1	1.6
Others	10	16.4
Taking medicine(s) for chronic disease(s)		
Yes	36	59.0
No	25	41.0

**Table 3:** Dietary supplement usage among students.

	Frequency	Percentage	Frequency and Percentage of Dietary Supplements Usage			
			No		Yes	
			f	%	f	%
Students with chronic diseases	61	9.4	50	82	11	18
Students with no chronic diseases	509	90.6	78	15	431	85

**Table 4:** Correlation between program and knowledge of the DSs.

	Chi^2^	*p*-Value	Alpha	Result
Program	119.458	<0.00001	0.05	Significant
knowledge of dietary supplements				

**Table 5:** Frequency and percentage distribution of behavior during the pandemic.

	Frequency	Percentage
Have your dietary habits changed since the beginning of the COVID-19		
Yes, it changed for the better	146	22.4
Yes, it changed for the worse	117	18
No, it has not changed	231	35.5
I cannot specify	157	24.1
Do you think that you need to improve your immunity by changing some unhealthy dietary habits?		
Yes	527	81
No	65	10
I do not know	59	9.1
Do you think that following healthy habits may reduce your chance of getting Coronavirus?		
Yes	333	51.2
No	184	28.3
I do not know	134	20.6

**Table 6:** Reasons for not taking dietary supplements during the COVID-19 pandemic.

Reasons	Frequency	Percentage
High price	96	21.7
I do not know which of them is available in the market	101	22.9
They have some side effects	68	15.4
I cannot take them regularly (cannot adhere)	129	29.2
I do not need them, I do NOT have vitamin deficiency	77	17.4
I am committed to a healthy and balanced diet	24	5.4
They interact with my home medications	7	1.6
They are ineffective	10	2.3
They are unsafe	14	3.2
There is no specific reason	163	36.9

**Table 7:** The Dietary supplements.

	Count	Percentage
Which of these dietary supplements do you TAKE during COVID-19 outbreak? (Select all that apply)		
Vitamin C	167	79.9
Vitamin D	75	35.9
Vitamin B Complex	10	4.8
Vitamin B12	18	8.6
Folic acid	13	6.2
Multivitamins	28	13.4
Zinc	40	19.1
Iron	65	30.1
Magnesium	13	6.2
Fish oil (omega-3 oils)	21	10
Indian costus	9	4.3
Sumac	9	4.3
Black seed	60	28.7
Garlic	47	22.5
Honey	99	47.4

**Table 8:** Reference for dietary supplements.

Who Suggested You Take Dietary Supplements?	Count	Percentage
A physician (a doctor)	40	19.1
A pharmacist	12	5.7
A nutrition specialist	5	2.4
A nurse	2	1
Social media/Internet	35	16.7
Health news on TV	4	1.9
Outreach awareness fliers	4	1.9
A friend/relative	44	21.9
Personal judgment or previous experience with the benefits of dietary supplements	57	27.3

## Data Availability

Not applicable.
